# Supplementary material for: Identification of MicroRNAs Associated with Histological Grade in Early-Stage Invasive Breast Cancer
Source: Int J Mol Sci. 2023 Dec 19;25(1):35. doi: 10.3390/ijms25010035 (PMC10779190; doi:10.3390/ijms25010035)
Supplement: Supplementary file 1 [file ijms-25-00035-s001.zip › ijms-2761464-supplementary.pdf]

Table S1. List of 108 microRNAs associated with breast cancer.

**hsa-let-7c**	**hsa-mir-139**	**hsa-mir-182**	**hsa-mir-26a-1**	**hsa-mir-34a**	**hsa-mir-424**	**hsa-mir-574**	**hsa-mir-944**
**hsa-let-7g**	**hsa-mir-142**	**hsa-mir-183**	**hsa-mir-299**	**hsa-mir-3607**	**hsa-mir-425**	**hsa-mir-582**	**hsa-mir-96**
**hsa-mir-100**	**hsa-mir-143**	**hsa-mir-187**	**hsa-mir-301a**	**hsa-mir-361**	**hsa-mir-4326**	**hsa-mir-585**	**hsa-mir-99a**
**hsa-mir-101**	**hsa-mir-144**	**hsa-mir-191**	**hsa-mir-301b**	**hsa-mir-3615**	**hsa-mir-449b**	**hsa-mir-589**	
**hsa-mir-10b**	**hsa-mir-145**	**hsa-mir-193a**	**hsa-mir-30e**	**hsa-mir-3677**	**hsa-mir-449c**	**hsa-mir-592**	
**hsa-mir-125b-2**	**hsa-mir-146b**	**hsa-mir-195**	**hsa-mir-3176**	**hsa-mir-3690**	**hsa-mir-452**	**hsa-mir-615**	
**hsa-mir-126**	**hsa-mir-147b**	**hsa-mir-19b-1**	**hsa-mir-3180-1**	**hsa-mir-374a**	**hsa-mir-454**	**hsa-mir-655**	
**hsa-mir-1260b**	**hsa-mir-148b**	**hsa-mir-200a**	**hsa-mir-3184**	**hsa-mir-376a-1**	**hsa-mir-455**	**hsa-mir-671**	
**hsa-mir-128-1**	**hsa-mir-151**	**hsa-mir-202**	**hsa-mir-324**	**hsa-mir-376b**	**hsa-mir-483**	**hsa-mir-7-1**	
**hsa-mir-1301**	**hsa-mir-152**	**hsa-mir-204**	**hsa-mir-330**	**hsa-mir-376c**	**hsa-mir-484**	**hsa-mir-766**	
**hsa-mir-1307**	**hsa-mir-154**	**hsa-mir-21**	**hsa-mir-331**	**hsa-mir-377**	**hsa-mir-486**	**hsa-mir-877**	
**hsa-mir-130a**	**hsa-mir-155**	**hsa-mir-210**	**hsa-mir-335**	**hsa-mir-378**	**hsa-mir-488**	**hsa-mir-891a**	
**hsa-mir-130b**	**hsa-mir-15b**	**hsa-mir-215**	**hsa-mir-339**	**hsa-mir-421**	**hsa-mir-497**	**hsa-mir-92b**	
**hsa-mir-133a-1**	**hsa-mir-181a-1**	**hsa-mir-218-1**	**hsa-mir-33b**	**hsa-mir-422a**	**hsa-mir-500a**	**hsa-mir-93**	
**hsa-mir-136**	**hsa-mir-181b-1**	**hsa-mir-224**	**hsa-mir-345**	**hsa-mir-423**	**hsa-mir-551b**	**hsa-mir-940**	

Table S2. Clinicopathological significance of histological grade in the present TCGA cohort.

Factors		Histological grade			Significance
		Low	High	Total	*p*-value
Age	> 60	142 (58.4%)	101 (41.6%)	243	0.62
	≤ 60	114 (61.0%)	73 (39.0%)	187	
Tumor size	pT2-4	176 (55.9%)	139 (44.1%)	315	0.011
	pT1	80 (69.6%)	35 (30.4%)	115	
Nodal status	Positive	138 (61.1%)	88 (38.9%)	226	0.62
	Negative	117 (58.5%)	83 (41.5%)	200	
ER	Positive	237 (72.7%)	89 (27.3%)	326	<0.0001
	Negative	15 (15.8%)	80 (84.2%)	95	
HER2	Positive	42 (59.2%)	29 (40.8%)	71	0.89
	Negative	188 (60.1%)	125 (39.9%)	313	

Abbreviations: ER: estrogen receptor, HER2: human epidermal growth factor 2.

Table S3. Clinicopathological significance of a subgroup based on microRNAs related to histological grade.

Factors		histological grade related microRNAs			Significance
		Subgroup 1	Subgroup 2	Total	*p*-value
**Histological grade**	**Grade 3**	**49 (28.2%)**	**125 (71.8%)**	**174**	**<0.0001**
	**Grade 1, 2**	**152 (59.4%)**	**104 (40.6%)**	**256**	
**Age**	**> 60**	**88 (47.1%)**	**99 (52.9%)**	**187**	**0.92**
	**≤ 60**	**113 (46.5%)**	**130 (53.5%)**	**243**	
**Tumor size**	**pT2-4**	**149 (47.3%)**	**166 (52.7%)**	**315**	**0.74**
	**pT1**	**52 (45.2%)**	**63 (54.8%)**	**115**	
**Nodal status**	**Positive**	**108 (47.8%)**	**118 (52.2%)**	**226**	**0.77**
	**Negative**	**92 (46.0%)**	**108 (54.0%)**	**200**	
**ER**	**Positive**	**172 (52.8%)**	**154 (47.2%)**	**326**	**<0.0001**
	**Negative**	**26 (27.4%)**	**69 (72.6%)**	**95**	
**HER2**	**Positive**	**27 (38.0%)**	**44 (62.0%)**	**71**	**0.067**
	**Negative**	**157 (50.2%)**	**156 (49.8%)**	**313**	

Abbreviations: ER: estrogen receptor, HER2: human epidermal growth factor 2.

Table S4. Clinicopathological significance of miR-3677 in the present TCGA cohort.

Factors		Expression of miR-3677			Significance
		Low	High	Total	*p*-value
Histological grade	Grade 3	53 (30.5%)	121 (69.5%)	174	<0.0001
	Grade 1, 2	162 (63.3%)	94 (36.7%)	256	
Age	> 60	124 (51.0%)	119 (49.0%)	243	0.70
	≤ 60	91 (48.7%)	96 (51.3%)	187	
Tumor size	pT2-4	156 (49.5%)	159 (74.0%)	315	0.83
	pT1	59 (51.3%)	56 (49.7%)	115	
Nodal status	Positive	115 (50.9%)	111 (49.1%)	226	0.85
	Negative	99 (49.5%)	101 (50.5%)	200	
ER	Positive	181 (55.5%)	145 (44.5%)	326	0.00011
	Negative	31 (32.6%)	64 (67.4%)	95	
HER2	Positive	21 (29.6%)	50 (70.4%)	71	0.00012
	Negative	173 (55.3%)	140 (44.7%)	313	

Abbreviations: ER: estrogen receptor, HER2: human epidermal growth factor 2.

Table S5. Clinicopathological characteristics of the 15 breast cancer cases

**Age range in year**	
59 and less	7
60 and over	8
**Tumor size**	
<2.0cm	2
≥ 2.0 cm	13
**Nodal status**	
Negative	10
Positive	5
**Nodal status**	
Negative	10
Positive	5
**Histological grade**	
Grade 1	0
Grade 2	3
Grade 3	12
**Molecular subtypes**	
ER-positive/HER2-negative	5
HER2-positive	5
Triple-negative	5
**Surgery (Breast)**	

Partial	4
Total	11
**Surgery (Axillary)**	
Sentinel lymph node biopsy	12
Axillary lymph node dissection	3

Figure S1. MA plot of miRNAs

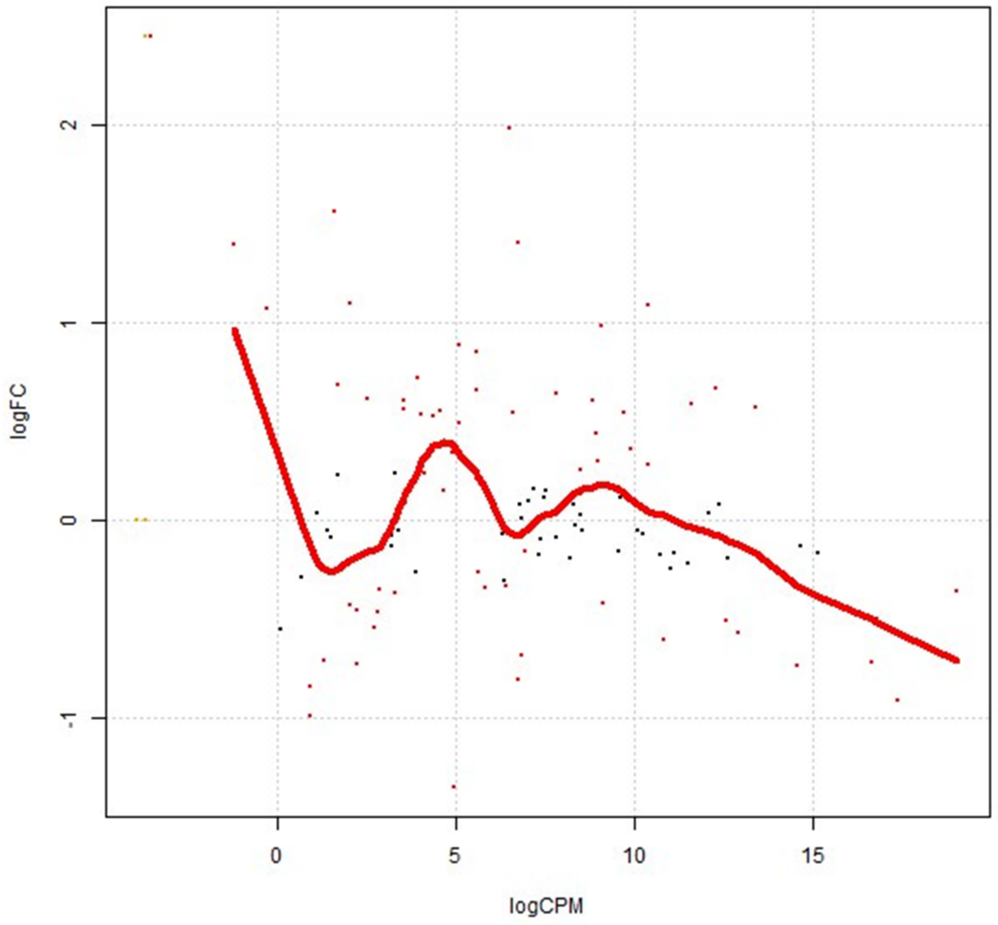

MicroRNAs that were called significantly differentially expressed are shown in red.
